# Dorsal and Ventral Hippocampus Modulate Autonomic Responses but Not Behavioral Consequences Associated to Acute Restraint Stress in Rats

**DOI:** 10.1371/journal.pone.0077750

**Published:** 2013-10-17

**Authors:** América A. Scopinho, Sabrina F. S. Lisboa, Francisco S. Guimarães, Fernando M. A. Corrêa, Leonardo B. M. Resstel, Sâmia R. L. Joca

**Affiliations:** 1 Department of Pharmacology, School of Medicine of Ribeirão Preto, University of São Paulo, Ribeirão Preto, São Paulo, Brazil; 2 Laboratory of Pharmacology, Department of Physics and Chemistry, School of Pharmaceutical Sciences of Ribeirão Preto, University of São Paulo, Ribeirão Preto, São Paulo, Brazil; 3 Center for Interdisciplinary Research on Applied Neurosciences (NAPNA), University of São Paulo, São Paulo, Brazil; Sapienza University of Rome, Italy

## Abstract

Recent evidence has suggested that the dorsal (DH) and the ventral (VH) poles of the hippocampus are structurally, molecularly and functionally different regions. While the DH is preferentially involved in the modulation of spatial learning and memory, the VH modulates defensive behaviors related to anxiety. Acute restraint is an unavoidable stress situation that evokes marked and sustained autonomic changes, which are characterized by elevated blood pressure (BP), intense heart rate (HR) increases, skeletal muscle vasodilatation and cutaneous vasoconstriction, which are accompanied by a rapid skin temperature drop followed by body temperature increases. In addition to those autonomic responses, animals submitted to restraint also present behavioral changes, such as reduced exploration of the open arms of an elevated plus-maze (EPM), an anxiogenic-like effect. In the present work, we report a comparison between the effects of pharmacological inhibition of DH and VH neurotransmission on autonomic and behavioral responses evoked by acute restraint stress in rats. Bilateral microinjection of the unspeciﬁc synaptic blocker cobalt chloride (CoCl_2,_ 1mM) into the DH or VH attenuated BP and HR responses, as well as the decrease in the skin temperature, elicited by restraint stress exposure. Moreover, DH or VH inhibition before restraint did not change the delayed increased anxiety behavior observed 24 h later in the EPM. The present results demonstrate for the ﬁrst time that both DH and VH mediate stress-induced autonomic responses to restraint but they are not involved in the modulation of the delayed emotional consequences elicited by such stress.

## Introduction

Stress has been consistently shown as a risk factor for the development of cardiovascular diseases. In animals, several responses have been traditionally associated with stressor exposure, including the neuroendocrine hypothalamo-pituitary-adrenocortical (HPA) axis activation by the release of glucocorticoids and adrenocorticotropin hormone- ACTH [1-3], the autonomic system, indexed by peripheral catecholamine release, heart rate, blood pressure, or core body temperature measurements [4-10], and several behavioral responses characterized by inhibition of locomotor activity, inhibition of feeding and drinking [2,7,11,12] and the anxiogenic effect observed in the elevated plus-maze (EPM) 24 h after the stress [9,13,14]

Besides the hypothalamus and brain stem, which are essential for autonomic and neuroendocrine responses to stress, higher cognitive areas of the brain that play a key role in memory, anxiety, and decision-making, may also participate in the modulation autonomic and behavioral responses to stress. It has been shown, for example, that the prefrontal cortex [15], the lateral septal area [9,16] and the bed nucleus of stria terminalis [17] play important roles in the modulation of the cardiovascular and emotional components of the stress reaction. Interestingly, the inactivation of the dorsal hippocampus blocks the cardiovascular, but not the behavioral responses to an aversive conditioned context (Resstel et al., 2008b), suggesting that the emotional and the autonomic components of the stress reaction could be dissociated within this brain region.

The hippocampus is a limbic structure that has been shown to be involved in the modulation of cognitive processes, such as in learning and memory [18-21] and in defensive behaviors related to anxiety and behavioral adaptation to stress [14,22,23]. Correlational and epidemiological studies have implicated stress in human disorders that involve the hippocampus, including dementia [24,25], depression [26,27], schizophrenia [28] and anxiety [29]. In addition, genetic predisposition accounts for only a fraction of diseases, such as Alzheimer’s, where hippocampal dysfunction is profound [30,31]. Aversive experiences, including stress, may contribute to human and animal disorders associated with major hippocampal dysfunction [32-34]. 

Recent behavioral, anatomical, and gene expression studies have suggested that the hippocampus would comprise two distinct subregions along the dorsoventral axis: the rostral/dorsal (DH) and the caudal/ventral (VH) zones. The DH, which corresponds to the posterior hippocampus in primates, would perform primarily cognitive functions, while the VH (anterior in primates) would be more closely associated to stress, emotion and affect regulation [35,36]. In accordance with that hypothesis, lesions of the DH, but not of the VH, impair behavioral performance in cognitive tests, while lesions of the VH, but not of the DH, attenuate stress responses and emotional behavior in tests predictive of anxiety-like behaviors [22,23]. Moreover, it has recently been shown by means of optogenetic techniques that the dorsal dentate gyrus control exploratory drive and encoding of fear memory whereas the ventral dentate gyrus has no effect on contextual learning but powerfully controls behavioral responses associated to anxiety (Keirbeck et al., 2013). However, it is important to note that experimental evidence have been mixed, with some studies reporting that the DH and VH are also able to play similar or even complimentary roles regarding cognition and affect, as an attempt to provide contextual specificity to the emotional system and help in the discrimination between ambiguous and emotionally charged information [37,38].

In addition to those cognitive and emotional functions, the involvement of the hippocampus in the regulation of cardiovascular system has also been described. Ruy and Neafsey reported that electrical or chemical stimulation of dorsal or ventral hippocampus, with L-glutamate (L-glu), decreased heart rate, blood pressure and breathing rate, in unanesthetized rats [39]. Also, the inactivation of the DH blocks the cardiovascular, but not the behavioral responses to an aversive conditioned context in rats [40], suggesting that this structure play an important role on modulating cardiovascular responses generated by this aversive situation. Despite this aforementioned evidence, the involvement of the VH in the modulation of autonomic responses to stress remains to be investigated. In addition, there is no study aimed at comparing possible functional differences between VH and DH in the modulation of stress-induced autonomic responses, as well as stress-induced delayed emotional consequences. 

Acute restraint stress (RS) is a widely utilized experimental model to study the emotional and autonomic responses to stress. It is an unavoidable aversive situation where the animal is placed in a plastic tube or metal, which restricts its movements [41,42]. This stress model leads to hormonal changes [43], cardiovascular responses characterized by elevated blood pressure and heart rate [15,16] and skeletal muscle vasodilatation and cutaneous vasoconstriction, which are accompanied by a rapid skin temperature drop and followed by a rise in body temperature [9,44,45]. In addition to those autonomic responses, animals submitted to restraint also present behavioral changes, such as reduced exploratory activity in an open field [46-48], increased immobility in a forced swimming test [49] and reduced exploration of the open arms of an elevated plus-maze (EPM) [9,13,50]. Therefore, it is possible to evaluate the consequences of this stress model acutely by recording the autonomic responses during restraint session, and later, 24 h after the restraint session, by analyzing animals’ emotional state in the EPM.

Exposure to acute restraint stress induces the expression of the proto-oncogene c-Fos, a marker of neuronal activation, in several brain regions, including the hippocampus [51]. In addition, administration of glutamate NMDA receptor antagonists into the DH, immediately after RS, is able to attenuate the development of the stress-induced behavioral outcomes 24h later in the EPM [9,13,50] However, the involvement of the hippocampus in the modulation of acute autonomic responses to RS was not yet investigated. In addition, it has not been investigated whether there would be any functional difference between DH and VH in the modulation of autonomic and behavioral responses elicited by exposure to acute RS. Accordingly, the aim of this study was to evaluate the effects induced by acute reversible inactivation of the DH or VH neurotransmission by means of local microinjection of a nonselective neurotransmission blocker, cobalt chloride (CoCl_2_), in animals submitted to RS.

## Experimental Procedures

### 2.1 Animals

Male Wistar rats weighing 230–250 g were used. The animals were kept in the animal care unit of the Department of Pharmacology, School of Medicine of Ribeirão Preto, University of São Paulo. The rats were housed individually in plastic cages with free access to food and water under a 12 h light/dark cycle (lights on at 06.30 h). Experimental procedures were carried out following protocols approved by the Ethical Review Committee of the School of Medicine of Ribeirao Preto (protocol n. 150/2010), which complies with the guidelines laid down by the National Institutes of Health (NIH, Guide for the Care and Use of Laboratory Animals).

### 2.2 Surgery procedure

Five days before the experiment, the rats were anesthetized with 2,2,2-tribromoethanol (Sigma, St Louis, Missouri, USA) (250 g/kg, intraperitoneally). After scalp anesthesia with 2% lidocaine, the skull was surgically exposed and stainless steel guide cannulae (0.55 mm) were implanted bilaterally into the DH or VH using a stereotaxic apparatus (Stoelting, Wood Dale, Illinois, USA). Stereotaxic coordinates for cannula implantation in the hippocampus were chosen based on the rat brain atlas of Paxinos and Watson (1997): DH- AP: -4 mm from bregma, L: +2.6 mm from the medial suture, V: -2.1 mm from the skull, or VH- AP: -5 mm from bregma, L: +5 mm from the medial suture, V: -4 mm from the skull. The incisor bar position was set at +3.2 mm. Cannulae were ﬁxed to the skull with dental cement and one metal screw.

Twenty-four hours before the restraint stress (RS) session, the rats had a catheter (4 cm PE-10 segment heat-bound to a 13 cm PE-50 segment, Clay Adams, Parsippany, NJ, USA) inserted into the abdominal aorta through the femoral artery for blood pressure recording as described by 40. After each surgery, animals were treated with a polyantibiotic preparation of streptomycins and penicillins i.m. (Pentabiotico, Fort Dodge, Brazil) to prevent infection and with the nonsteroidal anti-inflammatory flunixine meglumine (2.5 mg/kg s.c.; Banamine, Schering Plough, Brazil) for post-operative analgesia.

### 2.3 Drugs

The following drugs were used: CoCl_2_ (Sigma, St. Louis, MO), tribromoethanol (Sigma) and urethane (Sigma).

### 2.4 Acute Restraint

In the morning period (07–12 h), the animals were transported to the experimental room in their home cages. Mean arterial pressure (MAP) and HR were recorded with a HP-7754A ampliﬁer (Hewlett Packard, Palo Alto, CA) connected to a signal acquisition board (Biopac M-100, Goleta, CA) and were computer processed. After 15 min of baseline recording, rats received bilateral microinjection into the DH or VH through a dental needle (0.3 mm OD) introduced into the guide cannula. The injection needle was 1 mm longer than the cannula. Saline (vehicle, 0.9% NaCl) or CoCl_2_ (1 mM) was bilaterally microinjected in a ﬁnal volume of 200 nL into the DH or VH over a 10-sec period, using a 1-µL syringe (7001KH; Hamilton Co., Reno, NV). After microinjection, the injection needle was left in place for 30 sec before being removed to avoid reﬂux. Ten minutes later, rats were submitted to a 60-min restraint period in a plastic cylindrical restraining tube (diameter 5 6.5 cm and length 5 15 cm). After restraint, the animals were returned to their cages. Each animal was submitted to only one restraint session.

### 2.5 Temperature measurements

Besides the cardiovascular parameters, variations in cutaneous temperature (CT) were recorded with the thermal camera Multi-Purpose Thermal Imager IRI 4010 (InfraRed Integrated Systems Ltd Park Circle, Tithe Barn Way Swan Valley Northampton, USA) at a distance of 50 cm. 

### 2.6 Elevated plus maze (EPM)

The EPM test was conducted as described before [50]. Briefly, the apparatus consisted of two opposite open arms (50 X 10 cm) crossed at a right angle by two arms of the same dimensions enclosed by 40 cm high walls with no roof. The maze was located 50 cm above the floor. Rodents naturally avoid the open arms of the EPM and anxiolytic compounds typically increase the exploration of these arms without changing the number of enclosed-arm entries [9,52]. The AnyMazeTM software (version 4.7, Stoelting) was employed for behavioral analysis. It detects the position of the animal in the maze and calculates the number of entries and time spent in open and enclosed arms.

### 2.7 Histological procedure

At the end of the experiments, rats were anesthetized with urethane (1.25 g/kg, i.p.) and 100 nL of 1% Evan’s blue dye was bilaterally injected into the DH or VH to stain the injection sites. The chest was surgically opened, the descending aorta occluded, the right atrium severed and the brain perfused with 10% formalin through the left ventricle. Brains were postfixed for 24 h at 4°C, and 40 mm sections were cut using a cryostat (CM-1900, Leica, Germany). Sections were stained with 1% neutral blue and injection sites were identified.

### 2.8 Data Analysis

All autonomic responses were continuously recorded for 15 min before and during the 60-min of restraint stress period. Data were expressed as means ±SEM changes (respectively MAP, HR or CT) and were sampled at 5 min intervals as a mean of the changes during each 5 min. Points sampled during the 15 min before restraint were used as control baseline value. The autonomic values changes during restraint were analyzed using two-way ANOVA with treatment as independent factor and time as repeated measurement factor. The basal values changes were analyzed before and after vehicle or CoCl_2_ administration by Student’s t Test. The percentage of entries (100 x open/total entries) and time spent in the open arms (100 x open/open+enclosed) of the EPM were calculated for each rat. These data, together with the number of enclosed arm entries, were analyzed by one-way ANOVA followed by Bonferroni’s post hoc test. Values of P<0.05 were taken as showing statistically significant differences between means.

## Results

**Figure 1:**
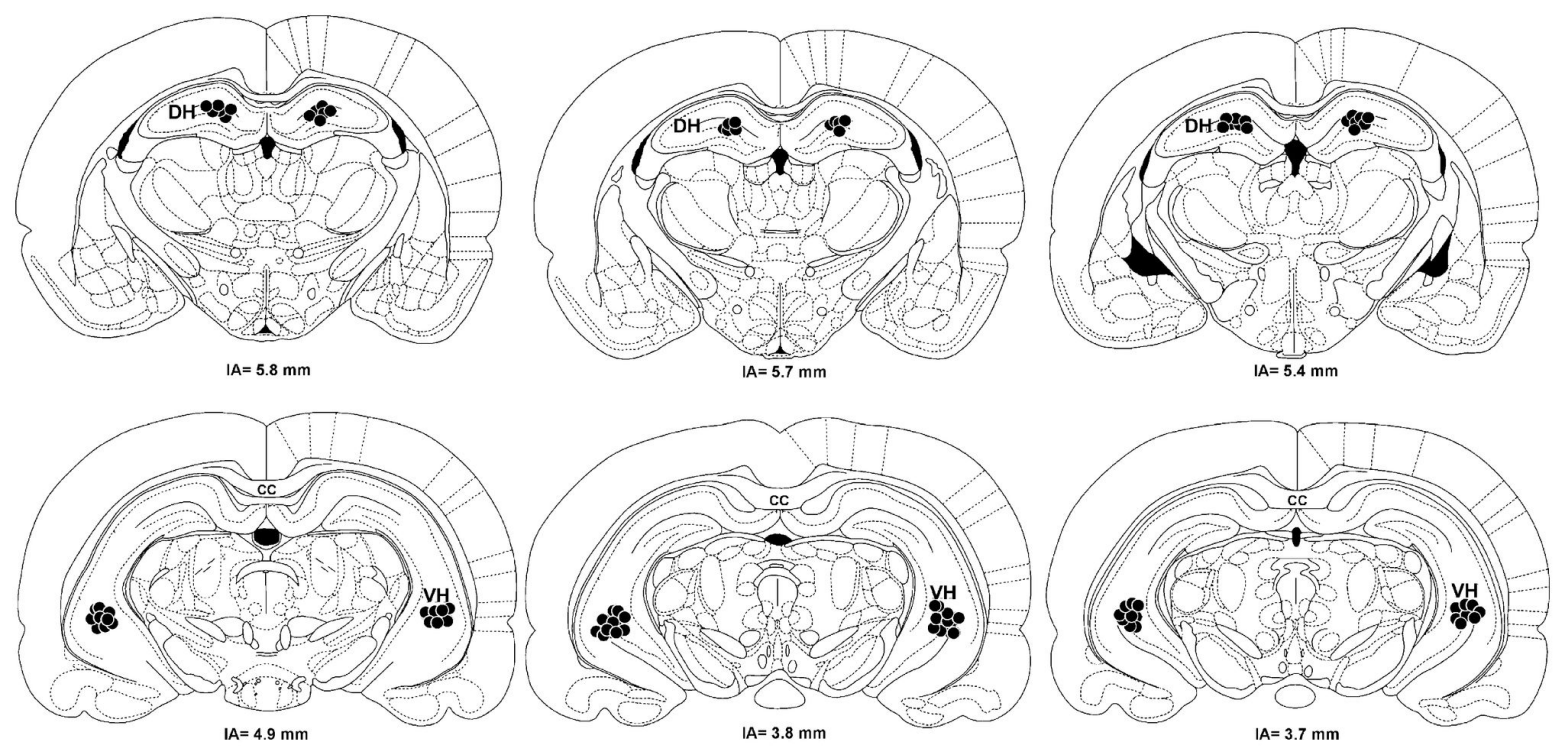
A diagrammatic representation based on the rat brain atlas of Paxinos and Watson (1997) indicating injections sites of vehicle or CoCl_2_ (closed circle) into the Dorsal Hippocampus (DH) and Ventral Hippocampus (VH). cc- corpus callosum; IA- Inter aural.

A representative diagrammatic representation indicating the injection sites into the DH and VH can be seen in Figure 1.

### 3.1 Effects of DH or VH inactivation on autonomic responses to acute restraint

The microinjection of CoCl_2_ into either the DH (n=6) or the VH (n=6) did not affect the baseline values of MAP (DH: F_3,20_ =0.06, P>0.05 and VH: F_3,20_ =0.06, P>0.05), HR (DH: F_3,20_ =0.4, P>0.05 and VH: F_3,20_ =0.4, P>0.05) or CT (DH: F_3,20_ =0.4, P>0.05 and VH: F_3,20_ =0.4, P>0.05). Acute restraint caused signiﬁcant increases in both MAP (F_14,165_=57.85, P<0.001), HR (F_14,165_=31.3, P< 0.001) and a significant and long-lasting decrease of CT (F_14,165_ = 20.11, P<0.001).

**Figure 2:**
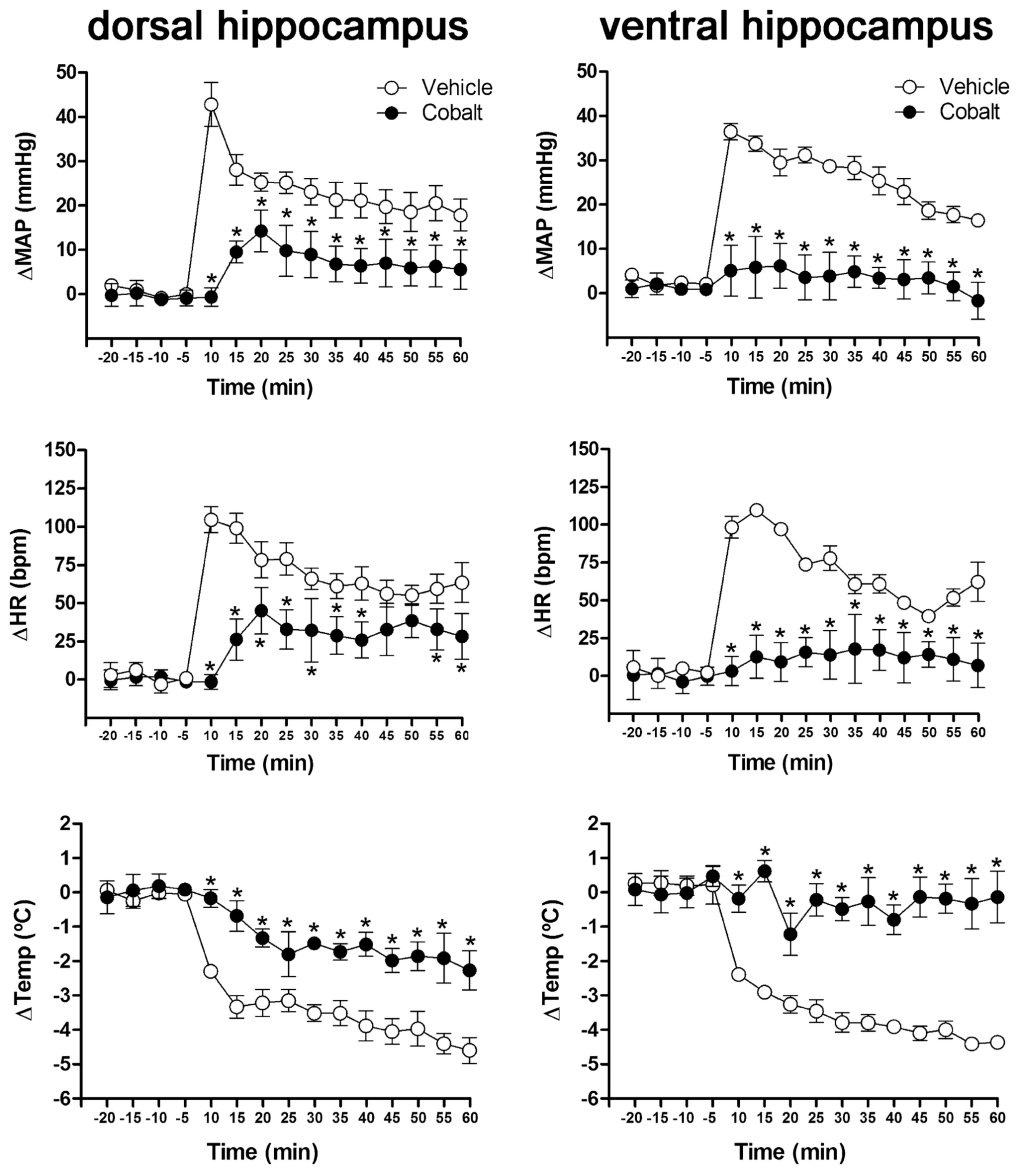
Time-course of bilateral microinjection of 200 nL of vehicle (n=6/ DH and VH) or 1 mM of CoCl_2_ (Cobalt, n=6/ DH and VH) administered into DH or VH on changes in mean arterial pressure (∆MAP), heart rate (∆HR) and cutaneous temperature (∆Temp) of animals submitted to 60 min of restraint stress. Symbols represent the means and bars the SEM. *P<0.05, Bonferroni’s post-hoc test.

**Figure 3:**
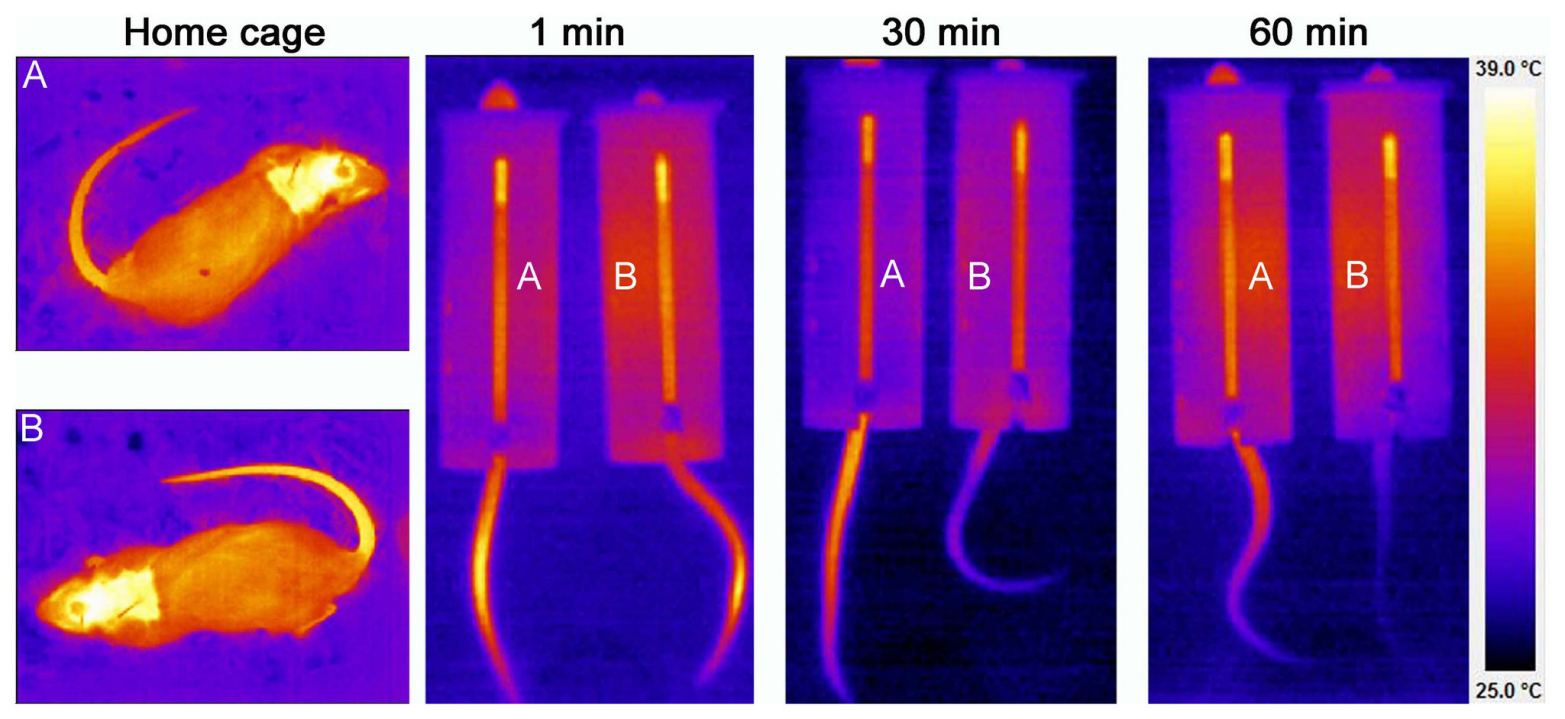
Infrared digital images of representative rats which received either CoCl2 (indicated by A) or vehicle (indicated by B) into DH, in its home cage and during the first minute, thirty and sixty minutes of restraint. Note the drop in cutaneous tail temperature during the restraint in vehicle treated animal and the absence of this drop in CoCl2 treated animal. The same effects were observed in VH treated animals. All images use the same color-coding for temperature.

The CoCl_2_ injection into the DH signiﬁcantly reduced MAP increases (F_1,165_=211.5, P<0.001), HR (F_3,165_=161.0, P<0.001), and blocked the fall in the CT evoked by RS (F_1,165_=124.7, P<0.001). In the same way, inhibition of VH with CoCl_2_ attenuated MAP (F _1,165_=89.5, P<0.01) and HR (F_1,165_=118.8, P<0.001) increase, and blocked the fall in the cutaneous temperature evoked by RS (F_1,165_=273.2, P<0.001) (Figure 2). Representative infrared digital images of representative rats that received either CoCl_2_ or vehicle into DH are presented in Figure 3.

### 3.2 Effects of DH or VH inactivation in the delayed anxiogenic effect induced by restraint exposure

**Figure 4:**
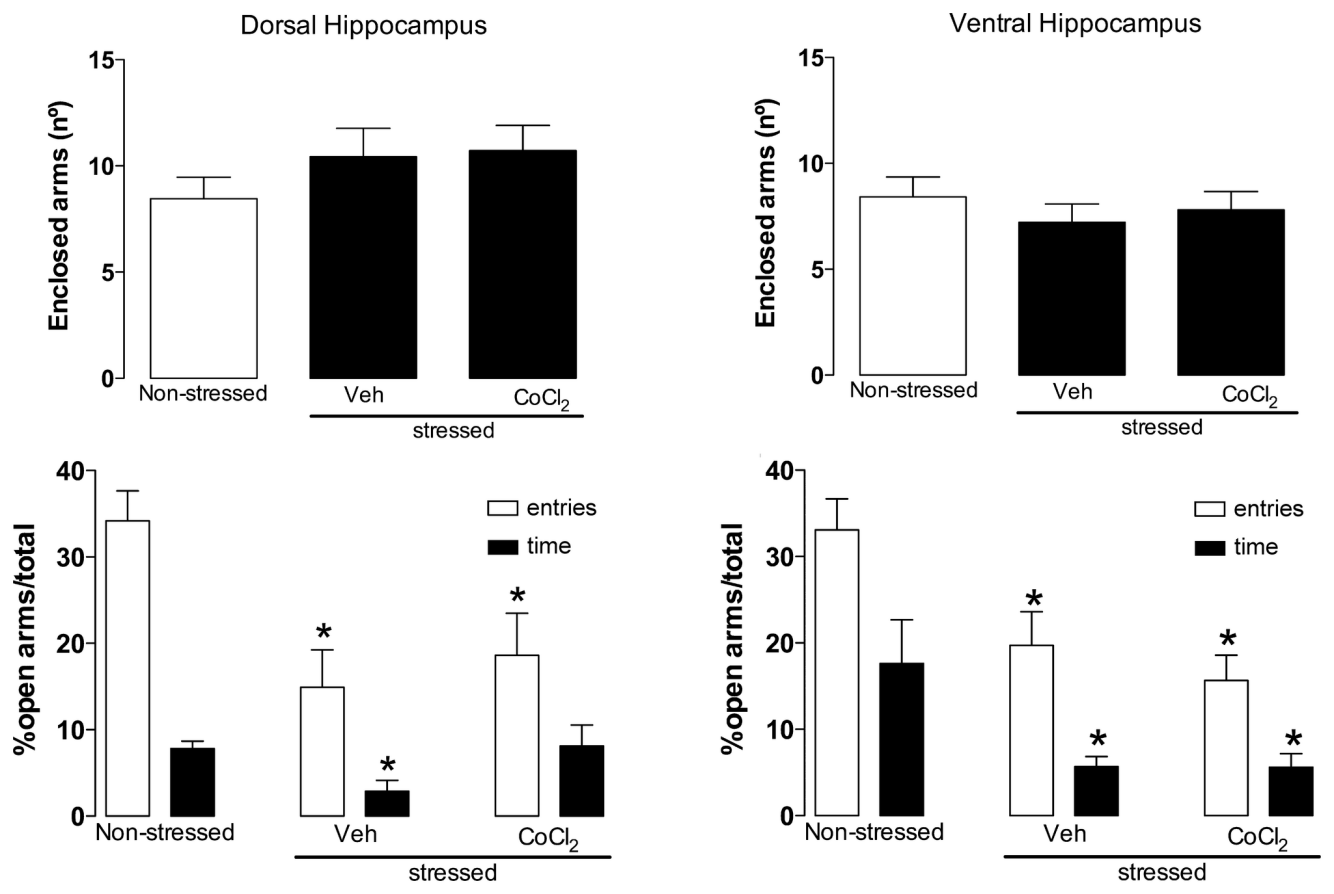
Effects of bilateral microinjection of 200 nL of vehicle (n=6/ DH and VH) or 1 mM of CoCl_2_ (n=6/ DH and VH) administered into DH or VH immediately before a 1-h restraint period on behavior observed 24 h later in the elevated plus-maze (EPM). A non-stressed group was used as control. Columns represent the means and the bars the SEM. *P<0.05, Bonferroni’s post-hoc test.

Animals submitted to acute restraint (n = 6) had a significant decrease in the percentage of time spent (F_2,17_ = 6.47, P<0.05) and in the number of entries in the open arms (F_2,17_ = 4.28, P<0.05) compared with unrestrained controls (n = 6). The CoCl_2_ administration into the DH (n= 6) or VH (n= 6), however, failed to change these effects (P>0.05, both) (Figure 4).

## Discussion

Acute restraint is an uncontrollable stress situation which produces several emotional and autonomic responses. The autonomic responses includes mean arterial pressure (MAP) and heart rate (HR) increases [16,17,53-55] skeletal muscle vasodilatation and cutaneous vasoconstriction, which are accompanied by a rapid skin temperature drop and followed by body temperature increases [44,45]. In addition to those autonomic responses, animals submitted to restraint also present behavioral changes such as reduced exploratory activity in an open field [46-48], increased immobility in a forced swimming test [49] and reduced exploration of the open arms of an elevated plus-maze (EPM) 24 h after the stress session [13,50]. Therefore, it is possible evaluated the consequences of this stress model acutely by autonomic responses during restraint session and later, 24 h after the restraint session, by anxiogenic like effect in EPM. Thus, our results showed that local neurotransmission within the DH and VH have similar inﬂuences on autonomic responses associated with acute RS. DH or VH neurotransmission inhibition reduced hypertension, tachycardia and skin temperature decreases elicited by RS exposure. However, local neurotransmission inhibition in the DH or VH before restraint did not change the delayed increased anxiety behavior observed 24 h later in the EPM, suggesting that this inhibition failed to prevent behavioral consequences of RS exposure.

Studies employing Fos protein/mRNA as a marker of neuronal functional activation have shown that exposure to stress, including restraint stress [51], promotes cellular activation in the hippocampus [56-58], therefore suggesting that this brain region is engaged in the modulation of stress reactions/responses. Our results are in agreement with that assumption, since the administration of the synaptic blocker CoCl_2_ into the VH or DH reduces the autonomic responses elicited by RS, showing that these hippocampal subregions may have similar roles in the modulation of autonomic responses associated with stress.

Microinjection of CoCl_2_ has been used by several researchers to promote the reversible inactivation of different brain nuclei, in order to understand their functional role [5,59-65]. CoCl_2_ evokes a reversible inactivation by reducing Ca^2+^ pre-synaptic influx and thus interfering with neurotransmitter release that leads to a synaptic blockage, without interfering with fibers of passage [66,67]. Thus, the use of CoCl_2_ causes temporary inactivation of local neurotransmission and, thus, minimizes several problems associated with lesion techniques. 

Corroborating our results, Resstel and co-workers demonstrated that the administration of CoCl_2_ into the DH attenuated the cardiovascular responses elicited by the re-exposure to an aversively conditioned context, but failed to modify its behavioral (freezing) consequences [40]. This finding suggests that the DH would play a preferential role in the modulation of the autonomic component of the stress response during an acute aversive situation. Similarly, Antoniadis and McDonald (2001) showed that hippocampal excitotoxic lesion with NMDA before conditioning did not modify freezing behaviour in the test session [68]. Together, these data suggest that the hippocampus appears to be important for autonomic modulation during aversive situations, although this structure has not yet received much attention in the field of research studying the central control of autonomic activity. 

Anatomical studies have indicated that the input and output connections of the DH and VH are distinct [69]. Spatial memory appears to depend on DH [70] while VH, but not DH, lesions alter stress responses and emotional behavior [71]. Lesions limited to DH evoked impairment on this task while lesions limited to VH resulted in enhanced learning [72].On the other hand, data of literature showed that either dorsal or ventral hippocampus NMDA lesions disrupt the rapid acquisition of new place information [37]. However, recently, the neuroanatomical bases for the functional differences found between the DH and VH were unclear. The DH present connections with bed nucleus of stria terminallis, lateral septal area and medial prefrontal cortex [69], structures that are known to modulate cardiovascular responses during RS [9,15-17]. The VH present bidirectional connectivity with basolateral, medial and central amygdalar nuclei [73-75]. Additionally, the VH and these amygdalar nuclei also share intimate bidirectional connectivity with the medial prefrontal cortex and agranular insular cortices [76-79] and also with bed nucleus of stria terminalis [36,80,81]. The bed nucleus of stria terminallis is one critical relay station for the hippocampal regulation of the hypothalamic-pituitary-adrenal response to psychological stress [82-84] and plays an important role in anxiety and cardiovascular regulation [17,85,86].

Although the DH and VH display distinctive patterns of connectivity and regulate several functions in different ways, both subareas of the hippocampus are connected with structures involved in cardiovascular modulation and it seems that both the DH and VH are similarly involved in modulation of cardiovascular responses caused by RS. Moreover, it has been previously suggested that the involvement of both DH and VH on the modulation of cardiovascular responses. Electrical and chemical stimulation of both VH and DH evoked marked decreases in heart rate and blood pressure [39]. In this situation, both VH and DH were able to similarly modulate cardiovascular responses. Therefore, it appears that both DH and VH are important for modulation of cardiovascular system. 

In agreement with the decreased cardiovascular responses to stress, VH and DH inhibition also reversed the stress-induced temperature changes. This finding indicates that during restraint stress both VH and DH could be modulating the activity of spinal sympathetic cardiomotor neurons and the sympathetic neurons controlling temperature changes. These latter neurons include those that control the cutaneous vascular tone in the tail skin. 

The cutaneous temperature in rats depends on the blood ﬂow and the sympathetic vasoconstrictor tone in the skin arteries [44,45]. Aversive situations cause a reduction in the tail blood ﬂow in rats [44,87]. The fall of the tail blood flow would occur to prevent blood loss due to injuries, by keeping low amounts of blood in the skin, and another function would be the redistribution of blood to more important organs during a stress situation. The cutaneous tail temperature can be used as an indirect measurement of blood ﬂow redistribution by the sympathetic nervous system in the rat [44,45,87]. Since that RS caused a fall in the tail temperature in control rats and pretreatment of the both DH and VH with CoCl_2_ inhibited the fall in tail temperature caused by the restraint, our results suggest that the hippocampus is an important modulator of the sympathetic tonus in the tail artery during the exposure to an aversive situation.

Acute restraint decreased the exploration of the open arms of the EPM 24 h after RS without changing the number of enclosed arms, corroborating with results in the literature [9,50]. The decreased exploratory activity is sensitive to systemic and intra-cerebral injection of anxiolytic and antidepressant drugs, suggesting that it reflects a delayed emotional effect induced by stress exposure [46-48,50]. In contrast with the results observed with autonomic parameters, DH and VH inactivation did not change the anxiogenic effects of RS. This result was not expected since that previous works showed that this structure plays an important role in the modulation of anxiety-related behaviors [22,88,89] and innate defensive responses to various threat stimuli [40,90-92]. The reasons for these contradictory results could involve the use of different pharmacological tools and experimental paradigms. For example, the fact that CoCl_2_ affects synapses terminations by reducing Ca^2+^ pre-synaptic influx and thus interferes with several neurotransmitter release, without a specific effect on one particular neural mechanism [66,67]. In addition, the experimental design of previous studies contrasts with the experimental design used herein, since we investigated the effects induced by DH or VH manipulation on the delayed emotional consequences induced by RS, 24h later. In fact, our data is supported by a previous report where it is described that the blockade of NMDA receptors in the DH, before the RS exposure, failed to attenuate the anxiogenic effect observed in the EPM 24h later [50]. However, when the NMDA antagonist was administered into the DH immediately after RS or before the EPM test, 24h later, then it was able to attenuate the stress-induced anxiogenic effect [50]. One possible explanation for such results is that, since the hippocampus would not be inhibited after RS exposure, the consolidation of the aversive memory of that situation would have allowed the development of its emotional consequences. In fact, there are reports suggesting that the hippocampus would have a pivotal role in mediating the consolidation of aversive memories and that its inhibition would favor behavioral adaptation to stress by inhibiting the consolidation of such memories, and consequently, of its emotional outcomes [93,94]. In agreement with that proposal, the DH inactivation with CoCl_2_, immediately after footshock stress was able to attenuate the expression of fear conditioning, 24h after such stress [40].

In summary, the present study examined the possible differential participation of DH and VH on autonomic and behavioral responses evoked by RS. It was demonstrated for the ﬁrst time that both, DH and VH, mediate autonomic responses associated with RS. On the other hand, DH and VH activity during an acute RS exposure does not contribute to the development of its delayed emotional consequences. Perhaps more signiﬁcantly, our findings provide further evidences that the autonomic and behavioral components of the stress response are dissociated within the DH and VH. 
